# Quantifying attentional modulation of auditory-evoked cortical responses from single-trial electroencephalography

**DOI:** 10.3389/fnhum.2013.00115

**Published:** 2013-04-04

**Authors:** Inyong Choi, Siddharth Rajaram, Lenny A. Varghese, Barbara G. Shinn-Cunningham

**Affiliations:** ^1^Center for Computational Neuroscience and Neural Technology, Boston UniversityBoston, MA, USA; ^2^Department of Biomedical Engineering, Boston UniversityBoston, MA, USA

**Keywords:** auditory attention, spatial attention, auditory event-related potentials, single-trial classification, brain−computer interfaces

## Abstract

Selective auditory attention is essential for human listeners to be able to communicate in multi-source environments. Selective attention is known to modulate the neural representation of the auditory scene, boosting the representation of a target sound relative to the background, but the strength of this modulation, and the mechanisms contributing to it, are not well understood. Here, listeners performed a behavioral experiment demanding sustained, focused spatial auditory attention while we measured cortical responses using electroencephalography (EEG). We presented three concurrent melodic streams; listeners were asked to attend and analyze the melodic contour of one of the streams, randomly selected from trial to trial. In a control task, listeners heard the same sound mixtures, but performed the contour judgment task on a series of visual arrows, ignoring all auditory streams. We found that the cortical responses could be fit as weighted sum of event-related potentials evoked by the stimulus onsets in the competing streams. The weighting to a given stream was roughly 10 dB higher when it was attended compared to when another auditory stream was attended; during the visual task, the auditory gains were intermediate. We then used a template-matching classification scheme to classify single-trial EEG results. We found that in all subjects, we could determine which stream the subject was attending significantly better than by chance. By directly quantifying the effect of selective attention on auditory cortical responses, these results reveal that focused auditory attention both suppresses the response to an unattended stream and enhances the response to an attended stream. The single-trial classification results add to the growing body of literature suggesting that auditory attentional modulation is sufficiently robust that it could be used as a control mechanism in brain–computer interfaces (BCIs).

## Introduction

Most human listeners are able to selectively attend to a target sound in a complex scene with relative ease. This ability depends on both sensory and cognitive processes, which interact to enable us to segregate competing streams, focus selective attention on an important target source, and recognize the target sound's content (Bregman, 1990; Wrigley and Brown, 2004; Shinn-Cunningham and Best, 2008; Lee et al., 2013). Though the specific mechanisms supporting these processes are not well understood, gross changes in neural activity due to attention can be observed in auditory-evoked event related potentials (ERPs) measured using electroencephalography (EEG; e.g., Hillyard et al., 1973; Hansen and Hillyard, 1980; Woldorff et al., 1987). Such studies find changes in the amplitude and shape of ERPs, suggesting that selective attention acts as a gain on neural activity, causing a relative enhancement of the representation of attended sensory inputs and a relative decrease in the representation of unattended or ignored inputs (Hillyard et al., 1998). A particularly salient effect of selective auditory attention is the enhancement of the N1 ERP component evoked by an attended sound (e.g., Hillyard et al., 1973), which, given its 100 ms latency (relative to stimulus onset) suggests it is generated in early auditory sensory cortex (Scherg et al., 1989). The idea that selective auditory attention strongly modulates the neural representation of sound in sensory auditory cortex is also supported by MEG studies (Woldorff et al., 1993; Alho et al., 2012; Ding and Simon, 2012) and fMRI data (Grady et al., 1997; Jäncke et al., 1999; Janata et al., 2002).

The current study explores how selective attention modulates ERPs evoked by competing musical streams. Listeners performed a “contour judgment” task that required them to focus attention on one of three simultaneous melodic contours and make judgments about the shape of the attended contour. This task mimics a real-world listening situation by requiring listeners to focus and sustain attention on a stream in order to analyze its content. We fit the EEG data as a scaled sum of the neural responses elicited by the individual streams played in isolation, allowing the scaling to depend on how a listener focuses attention. By finding the best scaling factors, or “attentional gains,” we quantified the amount of attentional modulation of the cortical response.

A number of existing brain–computer interfaces (BCIs) track changes in EEG signals corresponding to changes in how a user directs attention to visual objects (Kelly et al., 2005; Allison et al., 2010). Traditional ERP studies of auditory attention demonstrate task-related changes in the morphology of ERPs averaged over many trials (Hill et al., 2005). While such studies show that attention modulates the neural response, they do not test whether effects are strong enough or consistent enough that single-trial evoked responses can be used to deduce how attention is directed. To the degree that single-trial EEG classification is possible, it suggests that a BCI could be constructed that determines how a user (such as a locked-in patient) is focusing attention and then uses this information to navigate a command menu or control a device. A few recent studies suggest that auditory attention can modulate EEG responses sufficiently to be used in such a manner (e.g., Kerlin et al., 2010; Hill and Schölkopf, 2012; Lopez-Gordo et al., 2012). Because the modulation of attentional gain was pronounced in our experimental paradigm, we tested whether our single-trial EEG results could be used to classify the direction to which a listener was attending. We used a template-matching classification approach (Woody, 1967; Kerlin et al., 2010) to estimate from single-trial epochs which source the listener had attended on each trial. Classification rates were significantly above chance for all subjects. Given this success using single-trial non-invasive EEG, our results add to the growing body of literature demonstrating the potential for auditory selective attention to be used in EEG-based BCIs. Our approach models ERP waveforms as templates and uses a cross-subject validation to test classification performance; thus, our success suggests that an auditory attention BCI even could even be used successfully “out of the box,” without user-specific training of the EEG classifier.

## Materials and methods

### Subjects

Ten volunteers (two female, aged 21–34 years) participated in the experiments. All were right handed and had normal hearing. All provided written informed consent to an experimental protocol approved by the Boston University Institutional Review Board. Subjects were compensated at the rate $20/h for their participation.

### Task description

Subjects performed two types of trials: auditory-attention trials and visual trials (used as a control). In all trials, listeners were presented with three auditory streams, one from left of center, one from midline, and one from right of center (see below). In the auditory-attention trials, a visual cue presented at the start of the trial instructed listeners to shift attention covertly to either the left or right auditory stream (always ignoring the center stream) while maintaining visual fixation on a fixed dot on the center of the computer stream. At the end of the auditory-attention trial, they were asked to identify whether the attended tone sequence was ascending, descending, or zigzagging. In the visual trials, the visual cue at the start of the trial indicated that listeners should attend to a sequence of arrows presented at the fixation point. Just as in the attend-auditory trials, subjects identified whether the arrows changed direction from down to up, up to down, or zigzagged. Subjects were instructed to ignore the auditory streams during visual-trial presentations.

The acoustic streams were statistically identical in auditory-attention and in visual trials; only the task of the subject differed across trial types, and only the direction of the attended auditory stream differed between attend-left and attend-right auditory trials. In both auditory-attention and visual trials, subjects identified which kind of sequence was present by pressing one of three buttons (thus equating the motor planning and execution in the responses to the two trial types).

### Stimuli

**Figure 1:**
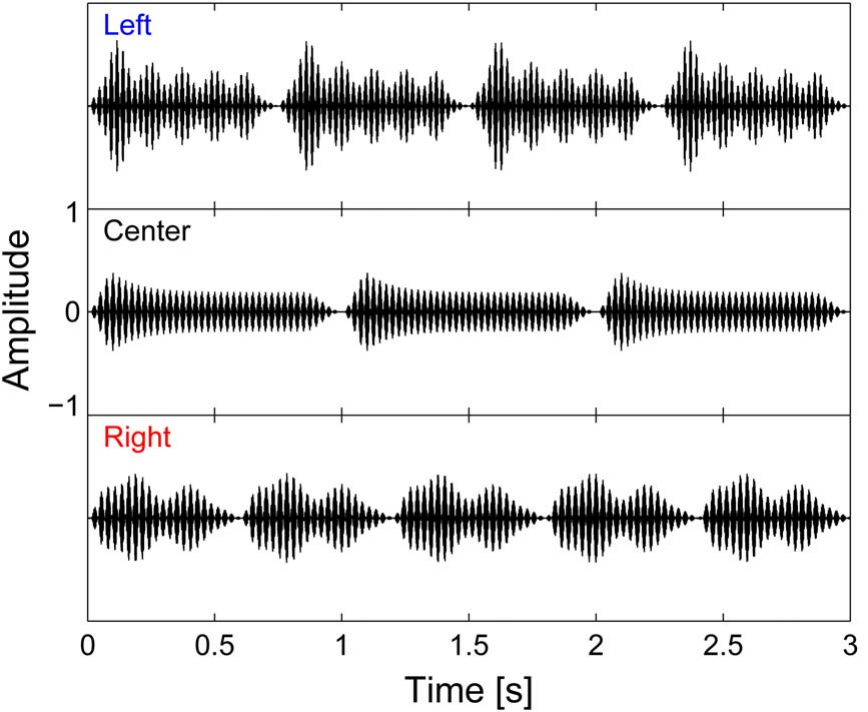
**Auditory stimuli waveforms.** Left, center, and right auditory streams are each isochronous, and made up of four, three, and five notes, respectively. All streams have a total duration of 3 s; however, except for the first note, the streams were designed to have note onsets that are temporally resolvable across streams note onsets are asynchronous.

All auditory stimuli were generated and processed using Matlab (Mathworks, Natick, MA). The auditory stimuli consisted of three concurrent melodic streams, each of which was comprised of multiple complex tones (henceforth referred to as “notes”). On each trial, each of the three streams had a distinct isochronous rhythm (three, four, or five notes), a distinct timbre (cello, clarinet, or oboe), a distinct pitch range that did not overlap with that of the other streams (bottom, middle, top), and a distinct lateral location (left, center, or right; see an example in Figure 1). This redundant set of cues ensured that the competing streams were easily segregated, perceptually, so that listeners could focus attention on whichever stream was important on a given trial.

The center stream, which was never the focus of attention, always consisted of three notes, each 1 s in duration; the left stream always contained four notes, each 750 ms in duration; and the right stream had five notes, each 600 ms in duration. All streams started and ended together and had the same total duration of 3 s. By design, although all three streams turned on together, each of the subsequent note onsets in each of the streams was distinct from the onsets in the other streams (see Figure 1). To achieve a natural, gradual time course, cosine squared onset and offset ramps (duration 100 ms) and a slowly decaying exponential curve (time constant 100 ms) were applied to each note.

The timbre and pitch range of the notes co-varied: the cello was always in the bottom pitch range, the clarinet in the middle pitch range, and the oboe in the top pitch range. To simulate the different timbres, we extracted the harmonic magnitudes of cello, clarinet, and oboe sound samples from a publically available corpus (Electronic Music Studios, University of Iowa, http://theremin.music.uiowa.edu/MIS.html) and applied these spectral shapes to synthetized harmonic complexes of the desired pitches (multiplying a broadband harmonic complex made up of equal intensity partials by the spectral filter derived from the appropriate instrument).

Each of the three instrument streams was made up of random sequences of two different notes, a low pitch and a high pitch, separated by roughly four semi-tones (a major third). The bottom-range cello notes had fundamental frequencies (F0) of either 240 or 300 Hz. The middle-range clarinet notes had F0s of either 320 or 400 Hz. The top-range oboe notes had F0s of either 720 or 900 Hz. In addition, the oboe stream was always played from the center (and hence was always to be ignored). In contrast, on each trial, the cello stream was randomly assigned to come either from the left (four note melody) or the right (five note melody), and the clarinet stream was assigned to come from the side opposite the cello stream.

The spatial directions of each stream were manipulated using head related transfer functions (HRTFs). HRTFs were recorded at a distance of 0.66 m in the horizontal plane, 0° in elevation, and at −60° (left), 0° (center), and +60° (right) in the azimuthal direction on a sample subject (methods described in Shinn-Cunningham et al., 2005).

The simple melodies making up each stream were constructed from the low and high notes of the associated instrument. On each trial, each melody was randomly selected to be ascending, descending, or zigzagging. For ascending trials, a transition point was selected randomly to fall anywhere between two notes, and then all notes prior to the transition were set to be low and all notes after the transition to be high. Similarly, for descending trials, a transition point was selected and all notes prior to the transition were set to be high and all notes after the transition to be low. Finally, zigzagging melodies was created by randomly selecting two transition points, then randomly setting all notes before the first transition to be either low or high, all notes between the transition points to be the opposite value (high or low) and all notes after the final transition point to be the same as the initial notes (e.g., valid five-note-long zigzagging melodies include L-L-H-H-L and H-L-H-H-H).

We were initially interested in whether attentional modulation could be observed in an auditory steady state response (ASSR) time locked to amplitude modulation of the notes in the streams, since the visual steady state response (VSSR) is a very effective marker of visual attention (Morgan et al., 1996; Müller et al., 1998, 2003; Ding et al., 2006). Therefore, the competing streams were also amplitude modulated at frequencies to which the EEG signal is known to phase lock in response to an isolated, modulated auditory stream (Rees et al., 1986; Linden et al., 1987; Ross et al., 2000). We added a modest, low-alpha-range sinusoidal amplitude modulation (5 or 8 Hz, 50% amplitude depth) as well as a gamma-range sinusoidal amplitude modulation (37 or 43 Hz, 100% amplitude depth) to both the left and right streams (the streams that could be the target of attention). Specifically, one of the two streams was modulated at 5 and 37 Hz, and the other at 8 and 43 Hz (randomly selected from trial to trial). The center stream was always amplitude modulated at 40 Hz with 100% depth. This modulation made the notes sound somewhat different from their original timbres, but did not interfere with either pitch perception or with the perceptual segregation of the competing streams. The ASSR in the raw EEG responses was statistically significant (i.e., the phase-locking to the ASSR frequencies was above the noise floor for most subjects and conditions); however, the strength of the ASSR proved to be unreliable as a marker of attention (the ASSR increased with attentional focus in some subjects, did not change significantly in some subjects, and decreased in some subjects). Therefore, we did not consider the ASSR further.

In visual control trials, a visual stream of arrows was presented from the center fixation point. Each of the visual streams consisted of an isochronous sequence of six down (∨) and up (∧) arrows. Because there was no visual gap between frames, only transitions from down to up or from up to down were perceived as new events. On each visual-task trial, one of ten possible visual sequences was randomly selected and presented. Three of the visual streams had a single transition of down to up (DDDDUU; DDDUUU; DDUUUU), three had a single transition of up to down (UUUUDD; UUUDDD; UUDDDD), and four had two transitions (DDDDUD; DDUUUD; UUUUDU; UUDDDU). Note that none of the visual sequences had a transition between the first and second arrows. Given the stimulus timings, 40% of the visual trials had a visual transition between the second and third arrows (which temporally aligned with the onset of second note in the center auditory stream), 20% had a visual transition between the third and fourth arrows (which temporally aligned with the onset of third note in the left auditory stream), 40% had a visual transition between the fourth and fifth arrows (which temporally aligned with the onset of third note in the center auditory stream), and 40% had a transition between the fifth and sixth arrows.

### Stimulus presentation and trial structure

**Figure 2:**
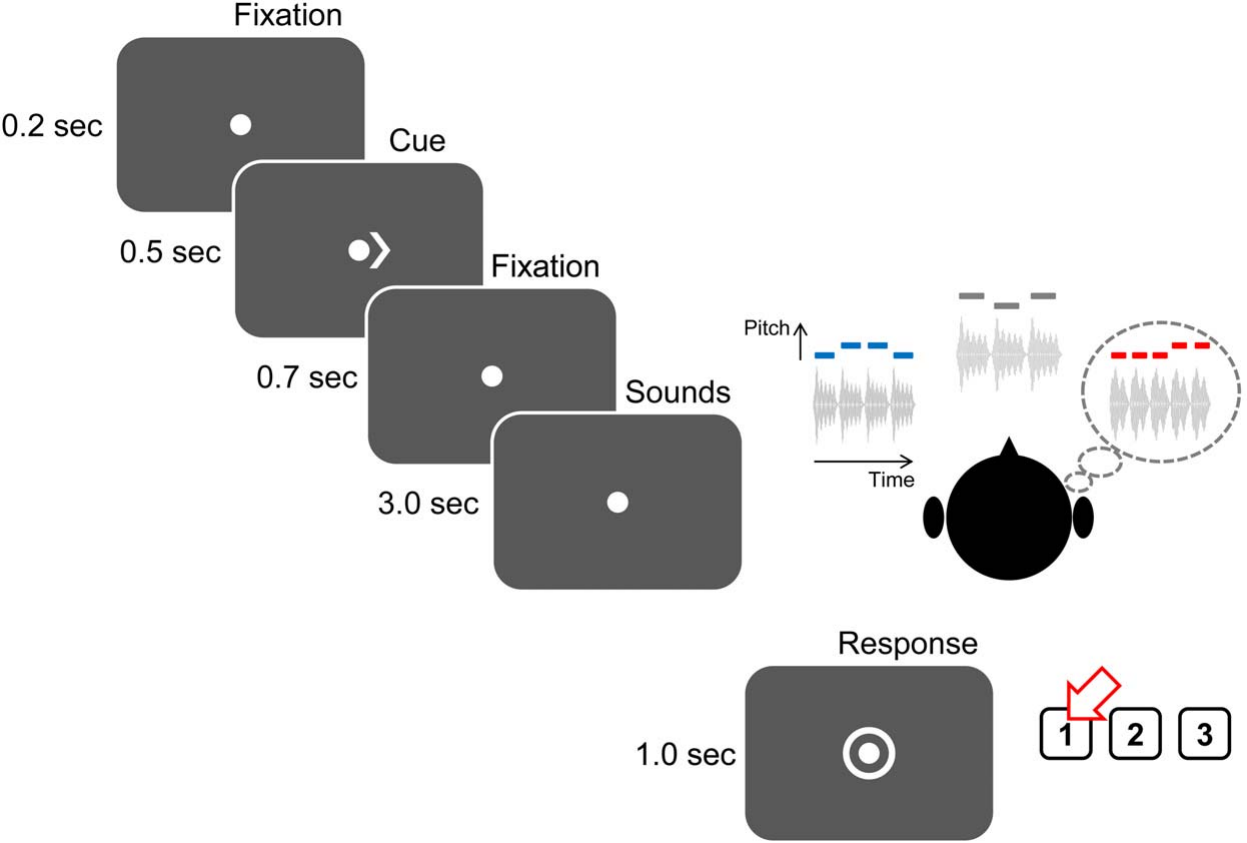
**Structure of an attend-auditory trial.** Trials begin with a visual fixation point presented for 200 ms, after which a 500-ms-long visual cue appears to instruct the subject as to what stream to attend (“<” to attend to the left auditory stream; “>,” as above, to attend to the right auditory stream; “< >” to attend to visual arrows appearing at the fixation point). There is a 700-ms-long gap before the sound (and, on attend-visual trials, arrow) streams begin to play. After the end of the streams, listeners have 1 s during which a circle surrounds the fixation point, instructing listeners to identify the kind of contour (“1” for ascending, as in the above example; “2” for descending; and “3” for zigzagging).

The experimental flow (illustrated in Figure 2) was controlled using Matlab with the Psychtoolbox 3 extension to present the visual cues and visual stimuli (Brainard, 1997; Pelli, 1997; Kleiner et al., 2007). Sound stimuli were presented using Etymotic (Elk Grove Village, IL) ER-1 insert headphones connected to a Tucker-Davis Technologies (Alachua, FL) System 3 unit. Software interfaced with the TDT hardware to play back the sound stimuli and to provide timing signals for EEG recordings. The stimulus sound level was fixed to 70 dB SPL, calibrated based on root-mean-squared values.

On each trial, subjects were instructed to fix their gaze to a dot presented at the center of the screen. After 200 ms, a visual cue appeared next to the dot and stayed on for 500 ms. This visual cue was either a single arrow (either “<” or “>”) indicating which auditory stream subjects should attend (auditory-attention trials) or two arrows facing opposite directions (“< >”) indicating a visual trial. 700 ms after the visual cue disappeared, the auditory stimuli described above were presented (duration 3 s). At the end of the sounds, a circle appeared around the fixation dot to indicate that the user should enter a response using a button box, either “1,” “2,” or “3” (corresponding to ascending, descending, or zigzagging). This response period lasted 1 s; answers made either before the sound finished playing or after the response period expired were marked as incorrect.

Subjects repeated training blocks of 20 auditory-attention trials until they achieved 80% or higher accuracy in labeling the melodies. Nine subjects were easily trained and achieved this threshold performance level within three demo sessions, but one subject failed to reach the 80% criterion even after repeated training blocks and was dismissed from further participation.

For the main portion of the experiment, a total of 480 trials were presented (12 blocks, each of 40 trials), with 320 auditory-attention trials (160 each for attend left and attend right), and 160 visual trials. Each of the 12 blocks contained roughly equal numbers of attend-left, attend-right, and visual trials, randomly ordered within a block.

### Passive single-note ERP measurement

To fit the cortical responses measured during the attend-auditory task, we used a single-note ERP elicited by the presentation of isolated complex tones. These results were used both in an ERP prediction model, and to compute weighting factors used in single-trial EEG classifications. Three of the 9 subjects participated in the single-note EEG measurement. Subjects watched a silent movie while listening to 100-ms long notes presented once a second for approximately 5 min, for a total of about 300 presentations per subject. The notes were comprised of the first 10 harmonics of 400 Hz, and had 5-ms-long cosine-squared on and off ramps.

### EEG data acquisition and analysis

EEG data was collected in all blocks of the behavioral task, as well as during the passive single-note ERP measurement. EEG data was collected using a Biosemi ActiveTwo system to record at 2048 Hz from 32 scalp electrode positions in the standard 10/20 configuration. Four additional electrodes monitored vertical and horizontal EOG, and two electrodes were placed on the mastoids for reference. Timing signals sent from the TDT to mark stimulus events were recorded in an additional channel. The recordings were re-referenced to the average of the two mastoid electrode responses, then bandpass filtered from 2 to 10 Hz using a 2048 point FIR filter applied offline.

For each trial, measurements from each electrode were baseline corrected using the mean value from −100 to 0 ms relative to stimulus onset. The raw data were down-sampled to a 64 Hz sampling rate prior to analysis. Any trials contaminated by artifacts (a signal exceeding a threshold of ±70 μV) were rejected from further analyses.

In the behavioral task, epochs from −100 to 3000 ms relative to the start of the first note were extracted. Each epoch was baseline to the mean of the pre-stimulus response (−100 to 0 ms). Any trials in which the subject responded incorrectly were removed from further EEG analysis. After removing trials with EEG artifact and with incorrect responses, there were a minimum of 244 (76%) trials and a maximum of 305 (95%) trials of each type of trial (attend-left, attend-right, visual task) for each of the subjects. ERPs for a given condition were first averaged for each subject, then averaged across subjects to yield the grand-average ERP.

Single-note ERP measurements were analyzed in epochs from −0.1 s to +0.5 s relative to the onset of the isolated note. After artifact rejection, the remaining data (714 trials, combined across the three subjects) was averaged to estimate a stereotypical single-note ERP. As discussed in the results, this single-note ERP estimate was used to model the ERPs evoked by individual notes in each stream during the attend-auditory task.

## Results

### Behavioral results

In general, subjects performed well on the behavioral task. Across subjects, mean performance was 95.9, 92.7, and 94.8% for attend-visual, attend-left, and attend-right conditions, respectively. Individual subject performance ranged from 99.1% correct (subject 8) down to 81.9% (subject 9) correct across the 9 subjects tested. There was no statistically significant difference in performance when listeners attended to the left (four-note) stream and when they attended to the right (five-note) stream (*t*-test, *p* = 0.21). Across subjects, performance in the attend-visual condition was significantly correlated with performance in the attend-auditory conditions (Pearson correlation coefficient *r* = 0.88, *p* = 0.0018). However, these differences in behavioral performance across subjects were not significantly correlated with any of the differences in the strength of attentional modulation that we found, discussed below (e.g., compared to the individual differences in the classification accuracy achieved, shown in Figure 9D, *r* = 0.52, *p* = 0.15).

### Single-note ERP

In order to model the effects of attention, we fit the ERPs in response to the three different attention conditions using approximations of the single-note ERP. Specifically, we first fit the single-note ERP with a simple, parameterized model, as described here, which we then used to model the full attend-auditory ERPs (see below).

**Figure 3:**
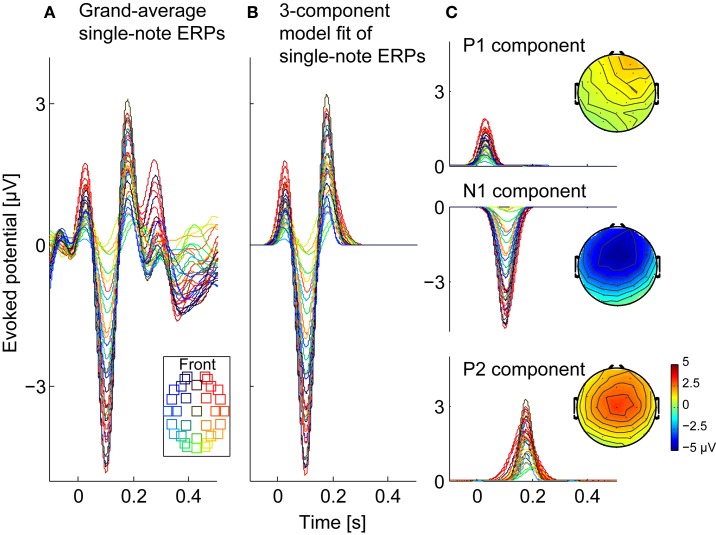
**Observed and modeled single-note ERPs. (A)** Observed single-note ERPs, averaged across three subjects (714 trials in total). Each trace shows responses from one of the 32 channels (see legend for electrode locations). **(B)** Three-component model of the single-note ERPs, fitted as a sum of three, scaled Gaussians (corresponding to P1, N1, and P2 components), with gains fit to the individual electrodes. **(C)** The three Gaussian components making up the 3-component model in **(B)** are shown separately, along with their scalp topographies.

The single-note ERP, shown in Figure 3A for all of the electrodes (different color traces), had prominent positive and negative deflections corresponding to a stereotypical ERP. The largest and most consistent of these deflections were the P1 (the first prominent positive reflection, around 50–80 ms post-onset), the N1 (the first negative deflection, around 100 ms post-onset), and the P2 (the positive deflection around 150–200 ms post-onset). Both the absolute magnitudes of these deflections and their relative strengths varied across electrodes, but tended to be strongest over frontal electrodes. Later negative and positive deflections were also present, but were smaller and more variable across electrodes.

At each electrode we approximated the first 250 ms of the single-note ERP using a three-component fit that had peaks corresponding to P1, N1, P2, each of which was modeled as a Gaussian function. Specifically, the 3-component fit of the single-note ERP for EEG channel k was given by:

(1) $$h_{k}^{\text{single}3}\left(t\right)=a_{k,P1}e^{-\frac{{\left(t-b_{k,P1}\right)}^{2}}{2c_{k,P1}{}^{2}}}-a_{k,N1}e^{-\frac{{\left(t\;-b_{k,N1}\right)}^{2}}{2c_{k,N1}{}^{2}}}+a_{k,P2}e^{-\frac{{\left(t-b_{k,P2}\right)}^{2}}{2c_{k,P2}{}^{2}}},k=\left\{1,2,\ldots ,32\right\}$$

Here, *t* represents time, while the parameters *a*_*k*, *i*_, *b*_*k*, *i*_, and *c*_*k*, *i*_ (*i* = *P*_1_, *N*_1_, *P*_2_) respectively determine the magnitude, peak response time (post-single-note onset), and duration of each of the three early ERP components measured at channel *k*. These parameters were fit using a non-linear least-squares method that iterated to find the best fit (based on the mean squared error) using the trust-region-reflective algorithm (Coleman and Li, 1996), with upper and lower bound constraints on *b*_*k*, *i*_ and *c*_*k*, *i*_ parameters. The upper and lower bounds were manually assigned based on the single-note ERP shown in Figure 3A; the lower bounds of *b*_*k*, *i*_ were 0, 73.2, and 161.1 ms and the upper bounds were 83.0, 156.3, and 244.1 ms for *i* = *P*_1_, *N*_1_, *P*_2_, respectively. Figure 3B shows the resulting *h*^single3^_*k*_(*t*), while Figure 3C shows the three constituent Gaussian components. As these results show, the dominant early features in the single-note ERP were well approximated by this simple fitting procedure.

### Attentional modulation of average ERPs

**Figure 4:**
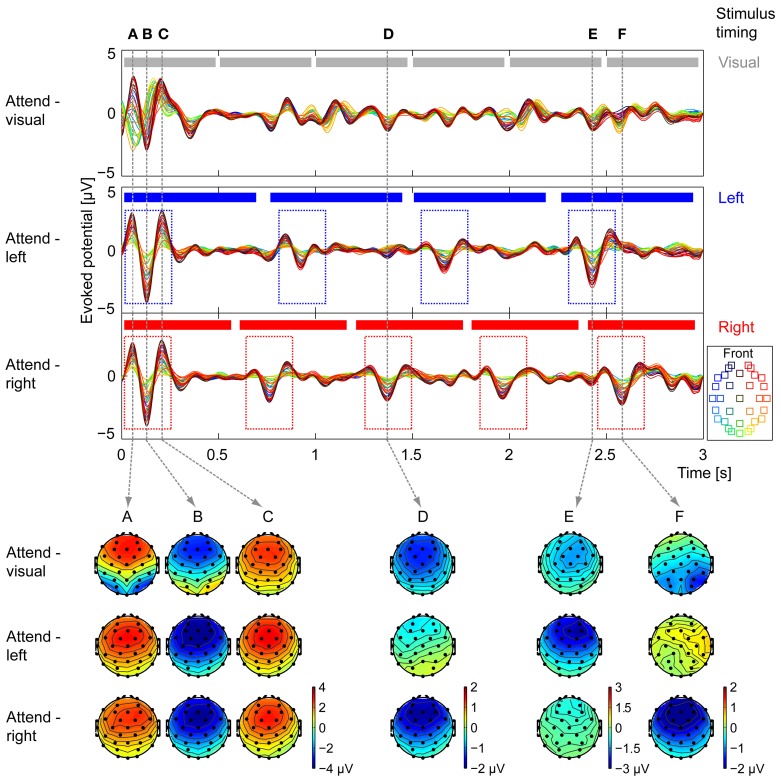
**Grand average ERP waveforms and topographies.** Average EEG waveforms evoked by the stimulus are plotted for each of the 32 electrodes (see legend for electrode location) in each of the attentional conditions (top: attend-visual, middle: attend-left, bottom: attend-right). At the top of each panel, the events in the attended stream are shown (gray, top panel: visual arrows; blue, middle panel: four notes of the left auditory stream; red, bottom panel: five notes of the right auditory stream). For attend-left and attend-right conditions, onset responses are seen following the note onsets of the attended stream (see dashed blue and red boxes in middle and bottom panels, respectively). The scalp topographies in each of the attentional conditions are shown for six key times, marked **(A–F)** by vertical dashed lines, that correspond to strong evoked responses in one or more conditions. **(A–C)** Times corresponding to auditory-evoked P1, N1, and P2 to the stimulus onset, respectively. Attend-left and attend-right conditions have nearly identical topographic patterns, with maximum amplitudes in frontal electrodes; in the attend-visual condition, occipital electrodes show a separate P1-N1-P2 pattern that leads the auditory responses. **(D)** Time of the N1 response to the third note of the right stream. Frontal electrodes show strong negative activity in the attend-right condition, moderate negative activity in the attend-visual condition, and weak activity in the attend-left condition. **(E)** Time of the N1 response to the fourth note of the left stream. Frontal electrodes show strong negative activity in the attend-left condition, moderate negative activity in the attend-visual condition, and weak activity in the attend-right condition. **(F)** Time of the N1 response to the fifth note of the right stream, as well as the response to the sixth arrow in the visual stream (only presented during attend-visual trials). The scalp distributions in the attend-right and attend-left conditions are similar to those from time point **(D)**. In the attend-visual condition, the strongest activity is over the occipital electrodes. EEGLAB (Delorme and Makeig, 2004) was used to generate the topographies.

Figure 4 shows grand-average ERPs for the visual-task, attend-left, and attend-right trials in three panels (top to bottom, respectively; within each panel, different colors correspond to different electrodes). At the top of each panel, the events in the attended stream are illustrated (six arrows, gray in top panel; four notes on left, blue in middle panel; five notes on right, red in bottom panel). For the stimulus mixture presented, the N1 responses evoked by visual-arrow transitions or acoustic note onsets should occur approximately 100 ms after the stimulus change, with a preceding positive deflection (P1) and a subsequent positive deflection (P2). Note that because the auditory stimuli were ramped to up with a 100-ms-onset window, the effective onset time to auditory events was shifted slightly later than the nominal onset of the stimulus.

At the expected time after the beginning of the trial, there was a very large ERP in all three attentional conditions. Importantly, this ERP had a noticeably different scalp distribution in the attend-visual and two attend-auditory conditions. In the attend-visual trials, the occipital electrodes, which typically respond strongly to visual stimuli (yellow-green traces; see legend), revealed large negative deflections prior to the large negative deflections in the frontal electrodes, which are known to respond robustly to auditory stimuli (red-blue electrodes; this temporal offset) between the visual and the auditory evoked responses likely reflects the above-mentioned shift in the effective onset times of the auditory stimuli caused by our time windowing. In the two types of attend-auditory responses, where there were no visual arrows, the occipital electrodes had a very small magnitude response that was temporally aligned with the dominant frontal-electrode (auditory) ERP. This can be seen clearly in the scalp distribution of the ERPs taken at time points roughly corresponding to the P1, N1, and P2 peaks in response to the evoked auditory response at the stimulus onset (times A, B, and C; see the scalp plots corresponding to the appropriate dashed vertical lines at the bottom of Figure 4 for the three different attention conditions). These distributions were similar for attend-left and attend-right conditions (middle and bottom rows of scalp distributions at times A, B, and C), with maximal magnitudes in the frontal electrodes; the attend-visual distribution showed additional activity over occipital electrodes, corresponding to a slightly earlier visual ERP evoked by the initial arrow (top row of scalp plots at times A, B, and C). Since the onsets to all streams were roughly aligned in time at the start of the trial, it is impossible to separate responses to the different auditory streams from these results. Therefore, all subsequent analysis ignores this initial onset response.

We next focused on the ERPs in response to later visual transitions (changes in arrows), which are only present in the attend-visual condition. Importantly, the visual streams had only 1–2 perceivable transitions in a given trial, and these transitions occurred at different time points in different trials (0, 40, 20, 40, and 40% between the pairs of adjacent arrows, as discussed above). This explains why subsequent visual ERPs corresponding to arrow transitions were small in magnitude; their magnitudes reflect the percentage of trials in which a given transition was perceivable (none for transition 1–2; small for 3–4; and moderate for 2–3, 4–5, and 5–6). In addition, these visually evoked ERPs were strongest over the occipital electrodes (yellow-green traces in the top of Figure 4).

Finally, we considered the ERPs in response to notes in the left and right auditory streams. In all conditions, the same auditory stimuli were presented; however, the magnitudes of the note-onset-driven ERPs depended dramatically on the attentional condition. In general, responses to a given note onset were large when listeners attended to the stream containing that note, small when they attended to the opposite stream, and intermediate when they attended to the visual stream (ignoring both auditory streams). This can be seen most clearly in the grand-average ERPs in the two auditory-attention conditions. There were only three prominent ERPs after stimulus onset when listeners attend the left, four-note stream, each of which aligned with one of the note onsets in the left stream (see dashed blue boxes in Figure 4). Similarly, in the attend-right grand-average ERP, there were four prominent ERPS after stimulus onset, corresponding to the onsets in the right, five-note stream (see dashed red boxes in Figure 4). Another key point is that the most robust portion of the evoked ERPs is the N1 component; within each condition, the positive components, although present, were often smaller and more variable in magnitude than the corresponding N1 component.

The scalp distributions at key points in time further demonstrate that attention strongly modulates responses to note onsets. At time D, which corresponds to the expected time of an N1 response to a note from the right, responses were strongly negative in frontal electrodes during attend-right trials (bottom distribution at time D), nearly absent in attend-left trials (middle distribution at time D), and intermediate in attend-visual trials (top distribution at time D). At time E, which corresponds to the expected time of an N1 response to a note from the left, responses were strongly negative in frontal electrodes during attend-left trials (middle distribution at time E), nearly absent in attend-right trials (bottom distribution at time E), and intermediate in attend-visual trials (top distribution at time E). Finally, time F aligns with the expected time of an N1 response to a note from the right and is close to the expected time of an N1 response to the visual transition for arrows 5–6, perceptible on 40% of the attend-visual trials. At time F, the attend-right ERPs were similar to those at time D (compare bottom distributions at times D and F), revealing a strong response to the attended, right-stream auditory note; however, there was almost no response to the same stimuli during the attend-left condition (middle distribution at time F is similar to that at time D). In the attend-visual condition (top distribution at time F), the greatest activity was over the occipital electrodes, corresponding to visual transitions in some of the trials near that time.

**Figure 5:**
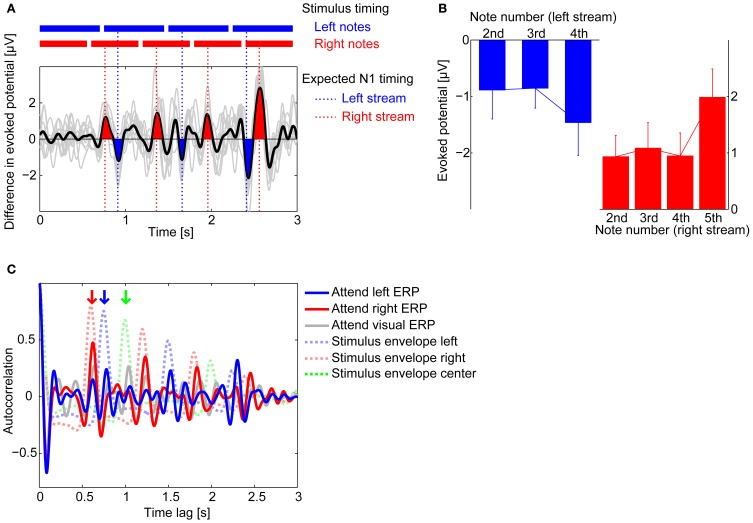
**Attentional modulation on averaged ERPs. (A)** Difference in the grand average ERP waveforms at Fz between attend-left (blue) and attend-right (red) conditions (attend left–attend right). Thin gray lines show the differences computed for each individual subject average ERPs, while black line shows the across-subject average. Deflections around the expected times of the N1 response to notes in the right stream are colored in red; deflections around the expected times of the N1 response to notes in the left stream are colored in blue. Results are consistent with attention accentuating the N1 response to notes in the attended stream relative to when that stream is ignored. **(B)** Across-subject means (± standard deviation) of the amplitudes of the difference waveform from **(A)** in electrode Fz, evaluated at the expected times of N1 responses to notes in the left stream (blue bar graph) and to notes in the right stream (red bar graph). **(C)** Auto-correlation functions (ACFs) of the grand average ERP waveforms measured at Fz (solid lines) and of the stimulus envelopes (dashed lines). The attend-left ERP ACF has a peak at 0.75 s, the inter-note interval in the left stream (red downward arrow); the attend-right ERP ACF has a peak at 0.60 s, the inter-note interval in the right stream (blue downward arrow); the attend-visual ERP ACF has peaks at the inter-note intervals of all three streams, including the center stream (green downward arrow).

Because stimuli were identical in the attend-left and attend-right conditions, another way to visualize the effects of attention is to subtract the ERPs in these two conditions. Figure 5A shows the difference in the ERPs for the two different auditory conditions, computed as the attend-left ERP–the attend-right ERP, averaged across subjects (note that if attention has no effect on the ERP, then this difference should be near zero; however, if attention to a stream leads to larger magnitude N1 deflections for onsets in that stream, this difference is expected to be positive at times corresponding to right-stream N1s and negative at times corresponding to the left-stream N1s). As expected, there were prominent positive peaks in this difference waveform at times corresponding to right-stream N1s (filled red in Figure 5A) and prominent negative peaks at times corresponding to left-stream N1s (filled blue in Figure 5A). To quantify the effects on the N1, Figure 5B plots the mean difference (error bars show the standard deviation across subjects) at the expected N1 position for notes 2, 3, and 4 in the left, four-note stream (blue bars) and for notes 2, 3, 4, and 5 for the right, five-note stream. These results confirm that the N1 had a significantly larger magnitude for the onsets in the attended stream than in the unattended stream (two-tailed *t*-tests on the values at these time points confirm that blue bars are all significantly smaller than zero and red bars are all significantly greater than zero, *p* < 0.01).

Consistent with this observation, both the attend-left and attend-right grand-average ERPs showed a periodic structure corresponding to the note onsets in the attended isochronous stream. To quantify this, we computed the auto-correlation function (ACF) of the attend-visual, attend-left, and attend-right grand-average ERPs, shown in Figure 5C (gray, blue, and red solid lines, respectively). For comparison, the ACFs of the stimuli envelopes of the left and right streams are shown as blue and red dashed lines in the same figure; the green dashed line shows the ACF for the center stream. Local ACF peaks occurred at 0.75 s for the attend-left condition and 0.6 s for the attend-right condition, which matched the inter-note repetition periods for the left stream and the right stream, respectively (shown by the blue and red vertical arrows at the top of Figure 5C); in these auditory-attention conditions, there was no prominent peak in the ACF at the repetition rates of unattended streams. Indeed, the peaks in the stimulus ACFs aligned closely with the peaks of the stimulus ACF when listeners were attending to that stream (compare dashed and solid lines of the same color in Figure 5C). Interestingly, in the attend-visual condition (gray solid line), there was a clear peak at the repetition period of the center stream, as well as peaks at the repetition periods of the left and right auditory streams (green, blue, and red vertical arrows, respectively).

### Fitting average ERPs and single-trial ERPs

**Figure 6:**
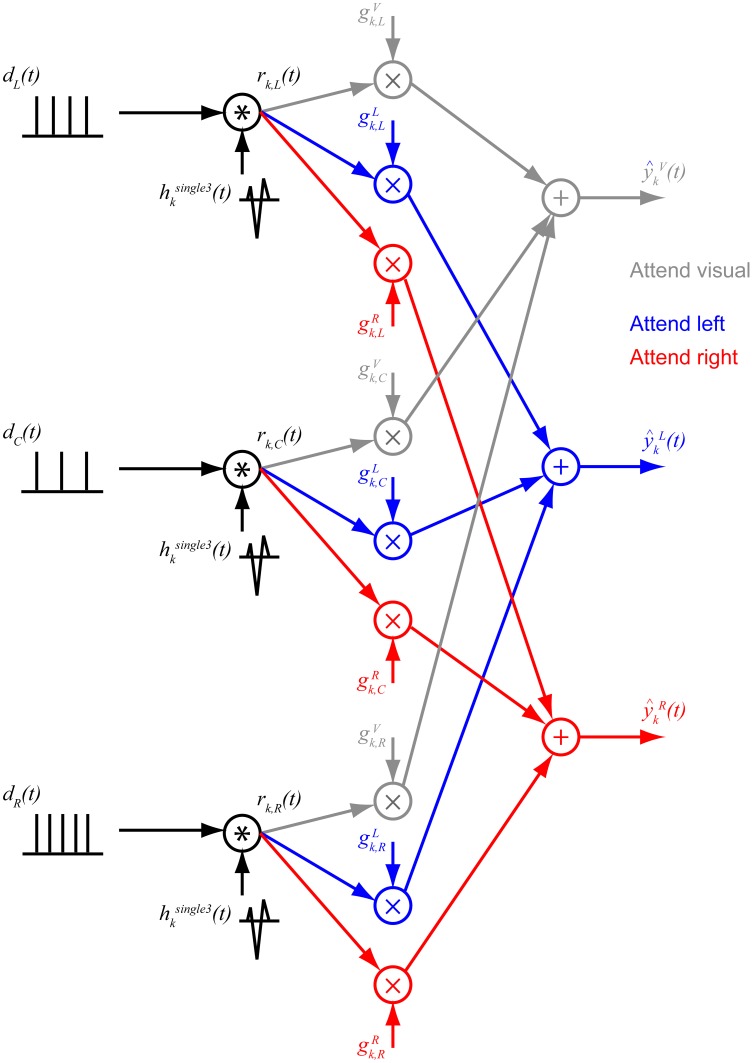
**Modeling ERPs.** Each stream is assumed to generate a sequence of onset responses corresponding to each note in the stream, the shape of which is equal to a modeled single-note ERP. The resulting signal from each stream is then scaled by a gain factor that depends on how attention is directed. These three stream-evoked signals are then summed to predict the total response in the three conditions (gray: attend visual; blue: attend left auditory stream; red: attend right auditory stream).

The single-note ERP *h*^single3^_*k*_(*t*) (described above) were used to fit the average attend-auditory ERPs for each subject by assuming that (1) the average attend-auditory ERP is a weighted sum of ERPs corresponding to each stream, (2) the ERP corresponding to a given stream is a superposition of single-note ERP evoked by the onsets of each of the notes in that stream, and (3) the relative strength of the ERP evoked by each stream is modulated by attention (see Figure 6 for a summary of the model). With these assumptions, each stream has a raw, unweighted contribution to the total ERP at electrode *k* that can be expressed as:

(2) $$r_{k,i}\left(t\right)=h_{k}^{\text{single}3}\left(t\right)\times d_{i}\left(t\right),\;i=\{L,C,R\}$$

where *d*_*i*_(*t*) is a train of impulse functions representing the note onset times for stream *i* (*L* for left, *C* for center, and *R* for right). To account for the ramped onset of the notes, the impulse function corresponding to each note in *d*_*i*_(*t*) was positioned 62.5 ms after the note onset. The raw stream ERPs *r*_*k*, *i*_(*t*) were then weighted, depending on the attention condition, to yield their contributions to the total attend-auditory ERP when attending the stream in direction *a*:

(3) $$s_{k,i}^{a}\left(t\right)=g_{k,i}^{a}r_{k,i}\left(t\right),\;a=\left\{L,R\right\}\text{ and }i=\{L,C,R\}$$

Then, the estimated total attend-auditory ERP at electrode k when attending to the stream in direction a can be written as:

(4) $$\begin{array}{l} \;{\hat{y}}_{k}^{a}\left(t\right)=\sum_{i=L,R,C}s_{k,i}^{a}\left(t\right)\\ \;={\vec{\vec{g}}}_{k}^{a}\times {\vec{\vec{r}}}_{k}^{T}\left(t\right)\;\\ \text{where }{\vec{\vec{g}}}_{k}^{a}=\left[g_{k,L}^{a},g_{k,C}^{a},g_{k,R}^{a}\right]\text{ and }{\vec{\vec{r}}}_{k}^{T}\left(t\right)\\ \;=\left[r_{k,L}\left(t\right),r_{k,C}\left(t\right),r_{k,R}\left(t\right)\right] \end{array}$$

The “attentional gains” in ${\vec{\vec{g}}}_{k}^{a}$ were fit by minimizing the mean square difference between the predicted total ERP and the measured ERP

(5) $$\underset{{}_{\left\{g_{k,i}^{a}\right\}}}{\min}\left\|{\hat{y}}_{k}^{a}\left(t\right)-y_{k}^{a}\left(t\right)\right\|,g_{k,i}^{a}\geq 0\text{ for }a=\left\{L,R\right\}\text{ and }i=\left\{L,C,R\right\}$$

where ‖ · ‖ denotes the L2 norm, and *y*^*L*^_*k*_(*t*) and *y*^*R*^_*k*_(*t*) are the measured ERPs in the attend-left and attend-right conditions, respectively. Note that the attentional gains {*g*^*a*^_*k*, *i*_} were constrained to be non-negative (see in Chen and Plemmons, 2007 for details of the method to add a non-negativity constraint to least square analysis).

For each subject, the mean attend-left ERP and the mean attend-right ERPs were fit (averaged over all correct-response, artifact-free trials) from 0.4 to 3.0 s after the onset of the first notes in the three streams. This fitting procedure ignored the first onset of the total ERP because (1) the model includes no adaptation effects, which cause the response to the first sound in a sequence of sounds to be larger than any subsequent sounds, and (2) the first onset is common to all streams, so it cannot isolate the response to each stream individually.

**Figure 7:**
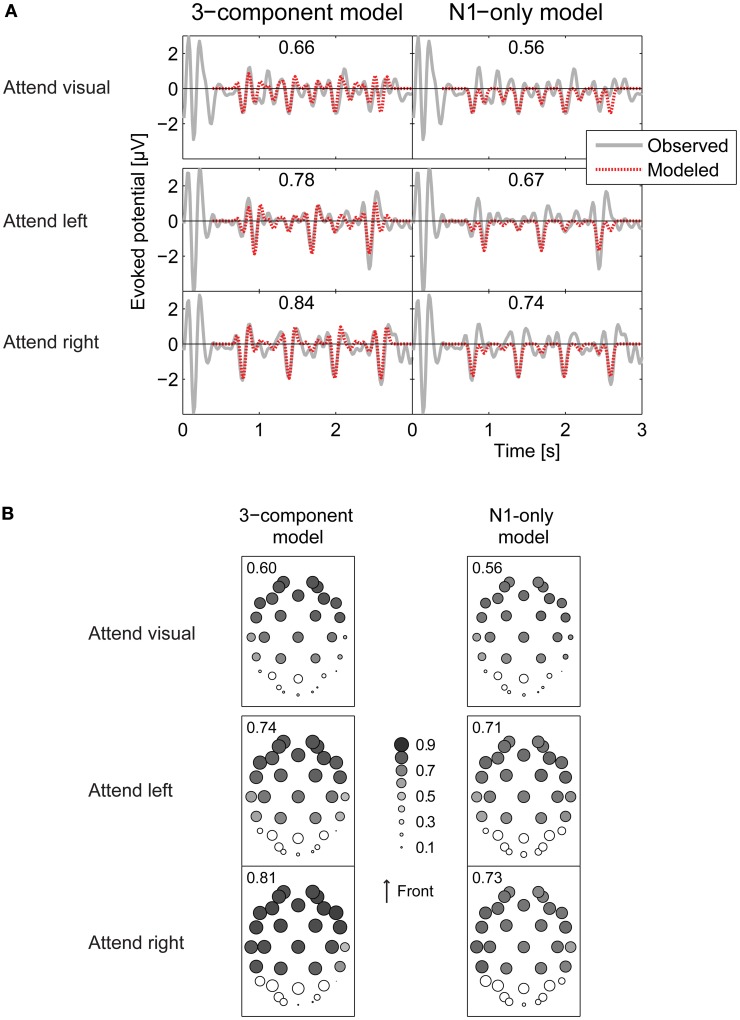
**Comparing observed and modeled ERPs. (A)** Observed (gray solid curve) and modeled (red dashed) ERP waveforms at the Fz electrode. Numbers in parentheses give the correlations coefficient between the observed and modeled ERPs. Top panels: attend-visual condition; middle panels: attend-left condition; bottom panels: attend-right condition. Left panels show the 3-component model fits; right panels show the N1-only model fits. Overall fits are better for the 3-component model than the N1-only model. Fits are generally better in attend-left and attend-right conditions than the attend-visual condition. **(B)** Correlation coefficients between observed ERPs and models at each of the 32 electrodes, represented by the diameter of the plotted circle. Correlations between observed and modeled responses are generally low over occipital electrodes (see open circles). Numbers in each panel give the average of the correlation coefficients over all electrodes except the occipital electrodes.

The pattern of results was very similar across subjects, so we focused on across-subject average results. The fitted average total ERPs closely corresponded to the measured ERPs at electrodes that respond robustly to auditory inputs. Figure 7A plot the across-subject average ERP at the Fz electrode (as an example), along with the average of the fitted ERP using Equation 4, for the attend-visual, attend-left, and attend-right conditions (top to bottom in left of Figure 7A, respectively). At the Fz electrode, correlation coefficients between the fitted waveforms averaged across all subjects and the measured grand-average ERPs were 0.66, 0.78, and 0.84 for the attend-visual, attend-left, and attend-right conditions, respectively. Conversely, at sensors where the response is not strongly driven by auditory stimuli, the fitted ERPs did not fit the measured responses as well. This can be seen in the left panels of Figure 7B, which show the correlation coefficients between the measured grand-averaged ERPs and the three-component model fit for all 32-channel electrode positions (the size of the circle over each electrode position denotes the size of the correlation coefficient). The correlations, averaged across subjects, ranged from 0.87 (at electrode F8 in the attend-right condition) down to 0.00 (at electrode P8 in the attend-left condition). In general, responses in the occipital electrodes, which tend to encode visual sensory responses robustly, were poorly fit by Equation 4, while the majority of the responses in more frontal electrodes were fit relatively well (in the left panels of Figure 7B, symbols are large in the frontal electrode positions, but small over the occipital positions at the bottom of the plots). Leaving out the occipital electrodes (show by open symbols in Figure 7B), the average correlation coefficients between modeled and observed ERPs (averaged over the remaining electrodes in each subject, and then averaged across all subjects) were 0.60, 0.74, and 0.81 for attend-visual, attend-left, and attend-right conditions, respectively. Standard deviations of the correlation coefficients across subjects were very small (0.03, 0.05, and 0.02 for attend-visual, attend-left, and attend-right, respectively), showing that the model captured similar effects in all subjects.

As already noted, the N1 response, the most prominent part of the total ERP, has previously been shown to be modulated by attention. To see how well an even simpler, N1-only model accounted for results, we reran all of the above analyses with *a*_*k*, *P*1_ and *a*_*k*, *P*2_ set to zero (only fitting the negative N1 deflection in the total ERP). Of course, the resulting fits captured fewer details in the total response. Even so, the simple fits still accounted for a significant portion of the variance in the responses. Specifically, at Fz, the correlations between these fits and the grand-average ERPs were 0.56, 0.67, and 0.74 for the attend-visual, attend-left, and attend-auditory conditions (see right panels of Figure 7A). Looking across all frontal electrodes (averaged across all subjects), the N1-only model of the ERPs yielded poorer overall fits than did the 3-component model, with correlation coefficients, from 0.77 (at electrode F4 in the attend-left condition) down to 0.06 (at electrode Oz in the attend-right condition). The average correlations across all frontal electrodes were 0.56, 0.71, and 0.73 for attend-visual, attend-left, and attend-right conditions, respectively. Again, standard deviations across subjects were very small (0.04, 0.03, and 0.02 for attend-visual, attend-left, and attend-right, respectively).

### Analysis of attention gains

**Figure 8:**
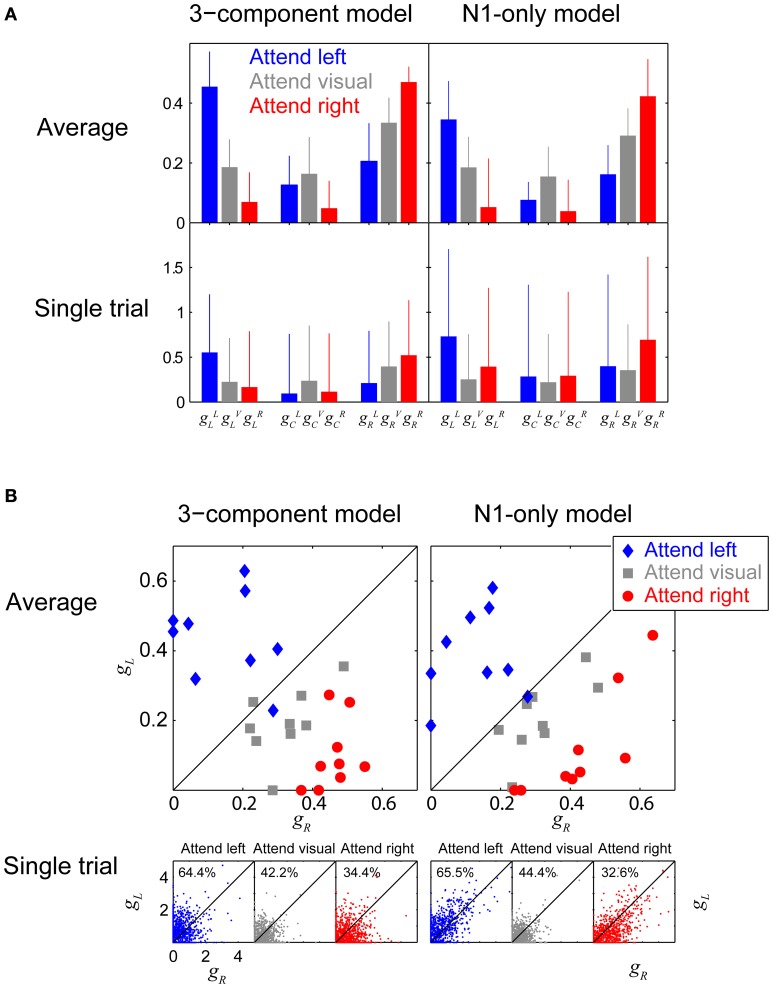
**Attentional gains. (A)** Attentional gains fit to Fz responses, obtained from both individual-subject average ERPs (top row), and single trials (bottom row). Left panels show gains fit using the 3-component model; right panels show gains fit using the N1-only model. The median values across subjects are plotted, with error-bars showing the root-mean-squared deviation from the medians. Blue, red, and gray bars represent attend-left, attend-right, and attend-visual conditions, respectively. Each triplet of adjacent bars shows the gains for one of the auditory streams (left, center, and right, going from left to right in a given panel). The gain to a given stream is greatest when it is attended, and smallest when a competing auditory stream is attended, and intermediate when the visual arrows are attended. **(B)** Attentional gains fit to the left and right streams (g_L_ and g_R_) obtained from individual-subject averages (top panels) and single-trial ERPs (bottom panels) in the three conditions (blue: attend left; gray: attend visual; red: attend right). In the single-trial panels, the numbers give the percentage of points falling above the diagonal. In general, the gain for the attended stream is greater than the gain for the ignored stream (blue points tend to fall above the diagonal and red points tend to fall below the diagonal).

To quantify how attention modulated responses, we analyzed the gains produced by the fitting procedure and compared them across conditions. Since the gains across the nine subjects were not Gaussian distributed (Shapiro-Wilk test rejected the hypothesis of normality), we computed the across-subject median (instead of the mean) and the root-mean-square-differences from the median (instead of the standard deviation). Figure 8A shows gain fits for the three-component model (left panels) and the N1-only model (right panels), computed both for across-trial-average ERPs, combined over subjects (top row) and the single-trial ERPs, combined over subjects and trials (bottom row). In each panel, each cluster of three adjacent blue, gray, and red bars represents the gains fit to one stream (left, center and right, from left to right). The color of each bar within a triplet denotes which attention condition these gains were derived from (blue: attend left, gray: attend visual, red: attend right).

The attentional focus had a statistically significant effect on the gains fit to the across-trial averaged ERPs. Specifically, the gain to the left stream was greatest when listeners attended that stream and smallest when they attended the right stream (the leftmost blue bar, ***g*^*L*^_*L*_**, is taller than the leftmost red bar, ***g*^*R*^_*L*_**, within each of the top two panels of Figure 8A; Wilcoxon rank sum test, *p* = 1.65 × 10^−4^ for the 3-component model and *p* = 0.0027 for the N1-only model). Similarly, the gain for the right stream was significantly greater when subjects attended to the right stream than when they attended to the left stream (***g*^*R*^_*R*_** > ***g*^*L*^_*R*_**, the rightmost red bar is taller than the rightmost blue bar in the top two panels of Figure 8A; Wilcoxon rank sum test, *p* = 4.11 × 10^−5^ for the 3-component model and *p* = 1.65 × 10^−4^ for the N1-only model). The gains were intermediate when subjects attended the visual arrows; for instance, using the N1-only model fits, the gain to the right stream was 1.45 times smaller (about −3 dB) in the attend-visual condition than in the attend-right condition and 1.8 times greater (about +5 dB) than in the attend-left condition. In the average-ERP results, the gain to the center stream, which is never the focus of attention, was larger during the attend-visual condition than the attend-left or attend-right conditions (in the center triplets of the top row of Figure 8A, the gray bars are taller than either the blue or red bars), although this difference was not statistically significant. Although the results were far more variable when the gain fit was done to single trial epochs, rather than to within-subject average ERPs, the same pattern of results was clear, showing that the attentional changes on ERPs is robust and strong (***g*^*L*^_*L*_** > ***g*^*R*^_*L*_**; Wilcoxon rank sum test, *p* << 0.001 for both the 3-component model and the N1-only model. ***g*^*R*^_*R*_** > ***g*^*L*^_*R*_**; *p* << 0.001 both for the 3-component model and the N1-only model).

Another way to examine the effect of attention is to directly compare the gain of the left and right streams for the same attention condition, rather than testing how attentional condition affected the gain to a given stream. This is done in Figure 8B, which plots *g*^*a*^_*R*_ against *g*^*a*^_*L*_ for attend-left, attend-visual, and attend-right conditions (*a* = *L*, blue points; *a* = *V*, gray points; and *a* = *R*, red points), both for the within-subject average ERPs (top panels; each subject contributes one point to each condition) and single trials (bottom panels; each subject contributes hundreds of individual trial points in each condition). Results are shown both the 3-component model fit (left panels) and the N1-only model fit (right panels).

In general, when fitting the within-subject average ERP, the gain for the attended auditory stream was greater than the gain for the ignored auditory stream (for both models, 8 out of 9 blue points fall above the diagonal and 9 out of 9 points fall below the diagonal in the top two panels of Figure 8B); results when listeners ignored both streams (gray) were intermediate to the other conditions. These results suggest that the estimated left and right stream gains that fit the total ERP can be directly compared to determine which stream a listener is attending (i.e., on average a subject is likely attending the stream whose gain is greater).

Of course, for such an approach to be useful for BCI, it would have to be reliable on a single-trial basis. The bottom panels of Figure 8B show that when attending the left stream, the gain fit to the left stream on an individual trial was generally greater than the gain fit the right stream (the majority of the single-trial blue points in Figure 8B fall above the diagonal, showing that *g*^*L*^_*L*_ tends to be greater than *g*^*L*^_*R*_). Similarly, when attending the right stream, the gain fit to the right stream on an individual trial was generally greater than the gain fit the left stream (the majority of the red points in Figure 8B fall below the diagonal, showing that *g*^*R*^_*R*_ tends to be greater than *g*^*R*^_*L*_). In the attend-visual condition, the left-stream and right stream gains were roughly equal, and roughly half of the points fell above the diagonal. Perhaps more importantly, among the trials that had at least one non-zero gain (92% of all trials), 65.0% of all trials fit using the 3-component model could be correctly classified into “attend-left” and “attend-right” categories by simply comparing the fitted gains to each trial's raw ERP (i.e., in the bottom-left panel of Figure 8B, 64.4% of the non-zero blue “attend left” trial results fall above the diagonal and 65.6% of the non-zero red “attend right” trial results fall below the diagonal). If anything, the N1-only model yielded slightly better classification; 66.5% of the single trials would be classified correctly based only on determining whether the left fitted gain or the right fitted gain was greater.

### Template matching for single-trial classification

As another test of whether auditory attentional modulation could be used to drive an EEG-based BCI, we performed template-matching classification of single trials (Woody, 1967; Kutas et al., 1977; Kerlin et al., 2010). This approach compares the ERP elicited on each trial *j* to both an attend-left template and an attend-right template [*T*^*L*^_*k*_(*t*) and *T*^*R*^_*k*_(*t*), respectively] and classifies the state of the subject based on which template is a better match to the observed ERP for that trial. We first used templates derived from the measured grand-average ERPs when listeners attend left and attend right (e.g., see Figure 4A). Given the similarities across subjects, we decided to try a very conservative leave-one-out cross-subject validation technique, rather than fitting individual subject results; we averaged the attend-left ERPs and the attend-right ERPs from all subjects except those of the subject whose data were to be classified, creating a template that was independent of the data being classified and based on patterns that were consistent across subjects, rather than fit to idiosyncrasies of the listener whose data were to be classified. (Note that fitting the templates to each individual subject might yield even better results, but would require an interface that was trained to the individual user before being deployed). In addition to using the grand-average measured ERPs, we also tried the same leave-one-out cross-subject validation approach using templates based on modeled ERPs of attend-left and attend-right conditions [${\hat{y}}_{k}^{L}\left(t\right)$ and ${\hat{y}}_{k}^{R}\left(t\right)$, respectively, defined in Equation 4; i.e., $T_{k}^{L}\left(t\right)={\hat{y}}_{k}^{L}\left(t\right)$ and $T_{k}^{R}\left(t\right)={\hat{y}}_{k}^{R}\left(t\right)$], using both the 3-component model and the N1-only model.

For trial *j*, we computed at each electrode *k* the normalized cross-correlation function *NCF*^*L*^_*k*, *j*_ between the EEG response at that electrode and the attend-left template, *T*^*L*^_*k*_(*t*), over a time range of 400–2800 ms). We also computed *NCF*^*R*^_*k*, *j*_, the normalized cross-correlation function (NCF) between the observed response and the attend-right template, *T*^*R*^_*k*_(*t*). These NCFs are a measure of the match between the response on trial *j* and the expected responses in the two attention conditions:

(6) $$NCF_{k,j}^{i}\left(\tau \right)=\frac{\int_{t=400\text{ ms}}^{2800\text{ ms}}m_{k,j}\left(t\right)\times T_{k}^{i}\left(t+\tau \right)dt}{\sqrt{\int_{t=400\text{ ms}}^{2800\text{ ms}}m_{k,j}^{2}\left(t\right)dt\times \int_{t=400\text{ ms}}^{2800\text{ ms}}{\left(T_{k}^{i}\left(t\right)\right)}^{2}dt}},i=\{L,R\}$$

The first 400 ms were excluded in this computation because the first note onsets were synchronous across streams and thus do not provide strong information about which stream is attended. Conversely, the final 200 ms (2800–3000 ms) contains little information about note onsets and may also be contaminated by motor planning activity not related to attention. At each electrode *k*, we then found the maxima of the attend-left and attend-right NCFs over the range −50 ms≤ τ ≤ 50 ms (to allow for a small time jitter in the responses compared to the templates), then took the difference of these maxima:

(7) $$x_{k,j}=\text{max}{\left[NCF_{k,j}^{R}(\tau )\right]}_{\tau \;=-50\text{ ms}}^{50\text{ ms}}-\text{max}{\left[NCF_{k,j}^{L}(\tau )\right]}_{\tau \;=-50\text{ ms}}^{50\text{ ms}}$$

Finally, on each trial, we computed a weighted average of these differences over the electrodes to come up with a single template-matching decision variable:

$$D_{j}=\sum_{k=1}^{32}w_{k}x_{k,j}$$

**Figure 9:**
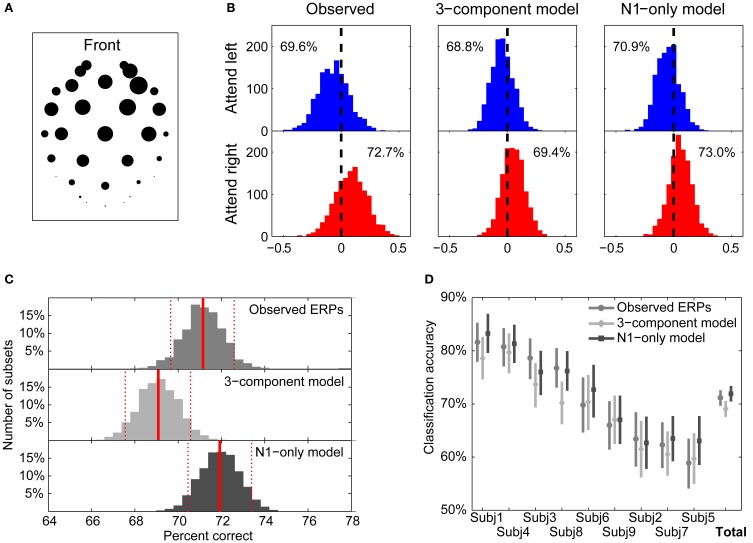
**Template-matching classification of single trials. (A)** Weighting factors for electrode locations obtained from principal component analysis on the measured single-note ERPs. The relative magnitude of the loading of each electrode is represented by the corresponding diameter of the circle. The greatest weights are given to mid-frontal channels. **(B)** Distributions of the template classification decision variable, *D*, for individual trials using templates from observed ERPs (left), the 3-component model (middle), and the N1-only model (right). Blue histograms plot the distributions for attend-left trials; red histograms plot the distributions for attend-right trials. The ratio of correctly classified trials (using zero as the decision boundary) is given within each panel. **(C)** Bootstrap distributions of classification accuracies using the three templates. Red solid lines represent the bootstrap means, while dashed lines represent 95% confidence intervals (CIs). Templates based on the 3-component model yield statistically worse classification performance than either templates equal to observed ERPs or templates based on the N1-only model. **(D)** Classification accuracies for individual subjects. Bootstrap means and 95% confidence intervals (CIs) are shown for each of the templates. For all subjects, performance is better than chance for all three template types.

With this processing, a negative value of *D*_*j*_ indicates that the attend-left template better matches the response on that trial (suggesting that the subject was attending to the left stream), while a positive value suggests that the subject was attending to the right stream. The weights {*w*_*k*_} were found by applying principal component analysis (PCA) to the single-note ERP results in the spatial domain (i.e., electrodes were treated as variables and the grand-average time domain data were treated as observations). The PCA produced a 32 × 32 matrix in which the rows are factor loadings for each electrode and the columns corresponded to the first 32 principal components, ordered based on how much of the variance could be accounted for with a particular distribution of activity over electrodes. In turn, the 32 loadings corresponding to a given principal component are weights reflecting the relative importance of each electrode to the corresponding principal component. The first principal component, which is the single spatial distribution that accounts for the greatest variance in the single-note ERP data, is shown in Figure 9A. The resulting weights were large for mid-frontal channels, where auditory evoked electric fields tend to be strongest, and lower over occipital channels.

Figure 9B shows the distributions of the *D*_*j*_ values for 1280 attending-left trials and 1343 attending-right trials, taken from all nine subjects. Results are shown using templates from the measured ERPs in attend-left and attend-right conditions (left panels), the 3-component model fits of the ERPs (middle panels), and the N1-only model fits (right panels). The top row shows the distributions of the decision variable for trials when listeners were attending the left; the bottom row shows the distributions of the decision variable when listeners were attending to the right. In general, the decision variable was more likely to be negative in attend-left conditions and more likely to be positive in attend-right conditions. The null hypothesis, that the distribution of *D*_*j*_ has a mean of zero, was rejected for all six of the distributions (two-sided *T*-tests; *p* < 0.001 for all six distributions). By comparing attend-left and attend-right distributions, we can compute the likelihood of correctly classifying an individual trial, assuming it is equally likely, a priori, that listeners are attending left or attending right. The corresponding classification probabilities were 71.2, 69.1, and 71.9% using the measured ERP templates, the 3-component model templates, and the N1-only model templates, respectively.

Although these overall classification accuracies were similar, we were interested in understanding whether the small observed differences were significant. A bootstrapping test was performed to compare these classification accuracies. (1) All 1280 + 1343 trials were randomly resampled with replacement (the Monte Carlo algorithm for case resampling: Manly, 1997). (2) The resampled trials were classified by using all the three templates to derive three classification accuracies were derived. (3) This process was repeated 2000 times to produce a bootstrap distribution of 2000 classification accuracies for each of templates. Figure 9C shows the resulting classification accuracy distributions using the three types of templates. The bootstrap means, indicated by solid red vertical lines, were almost identical to the observed means (71.1, 69.1, and 71.9% for the observed, 3-component model, and N1-only model ERPs, respectively). The distributions all were confirmed to be symmetrical about their means (Shapiro-Wilk test of composite normality; *p*-values are 0.31, 0.28, and 0.25 for the observed, 3-component model, and N1-only model, respectively). Since the distributions were centered and symmetrical, a simple percentile bootstrap method (Davison and Hinkley, 1997) was used to calculate the 95% confidence intervals (CIs) of the estimated classification accuracies; these are shown by dashed red vertical lines in Figure 9C. This analysis shows that classification performance using the N1-only model was statistically better than classification performance using the 3-component model (the means of each distribution fall outside the CIs of the other distributions). Similarly, the performance using the observed-ERP templates was statistically better than that using the 3-component-model ERP templates.

Finally, classification accuracies for the individual subjects are shown in Figure 9D for the three types of templates. Performance ranged from 82.9% (Subject 1 using the N1-only model ERP templates) down to 58.9% (Subject 5 using measured ERPs as templates). For each subject, the same bootstrapping approach was conducted to obtain distributions of classification accuracies for the three types of templates. For all subjects and all classification templates, classification performance was significantly greater than the 50% accuracy expected by chance (i.e., the lower bounds of the 95% CIs did not include 50%). Even though across all subjects the observed-ERP templates and the N1-only templates outperformed the 3-component templates, when considering the individual subject results, only Subjects 3 and 8 showed statistically significant differences consistent with the across-subject analysis.

## Discussion

### Quantifying auditory attention

When listeners need to analyze the spectrotemporal content of a sound source in the presence of simultaneous, competing sources, they must sustain selective attention on the target source. Our results show that in such situations, attention has a substantial effect on the sensory representation of a sound mixture in cortex. We quantified these effects by fitting gains to the ERP responses to attended and unattended auditory streams. We found that, on average, the best-fit gains to a stream changed by roughly 10 dB when that stream was attended vs. when it was ignored (Figure 8A). Moreover, this attentional modulation was sufficiently large and robust that the gains fit to single trial EEG differentiate which of two sound streams a listener is attending at rates better than chance (Figure 8B). These results show that attention causes a reliable change in cortical responses to identical sound stimuli that can be measured on the scalp using EEG.

Although a number of past studies have found that individual differences in behavioral ability correlate with individual differences in BCI control (e.g., Hill and Schölkopf, 2012), we found no consistent relationship. While there were consistent across-subject behavioral differences (e.g., performance on the visual task correlated strongly with performance on the auditory task), there was no consistent relationship between behavioral performance on the auditory task and the ability of a classifier to determine which acoustic stream a listener was attending. It may be that this lack of a relationship comes about because the factor limiting auditory task performance had little to do with the ability to direct selective auditory attention (which drives the classification performance), but rather reflected some other cognitive limitation common to the auditory and visual tasks. Of course, in general, all subjects performed the behavioral tasks very well. It may be that a behavioral task that is more sensitive to differentiating good and bad listeners would reveal a consistent relationship between behavioral ability and the ability to classify subject intention from EEG responses.

One interesting result of our study is that the cortical response to a particular auditory stream that a listener is ignoring were larger when the listener was attending to a visual stream than when he or she was attending to a competing auditory stream (for the average-ERP results in the top row of Figure 8A, gray bars are intermediate between blue and red bars for the left stream and the right stream, and above both blue and red bars for the center stream). This result suggests that focused auditory spatial attention not only enhances the representation of an attended auditory stream, but also suppresses the representation of an ignored auditory stream. Specifically, when a listener is focused on processing information in the visual modality, all of the auditory responses were greater than when listeners had to pick out one auditory stream from the acoustic mixture. Many psychophysical studies have suggested that there are both sensory-modality-specific resources as well as shared central resources when observers deal with competing sensory inputs (e.g., Alais et al., 2006; van Ee et al., 2009). Our results support the idea that when listeners are engaged in a visual task, they do not have to suppress the representation of incoming auditory streams in sensory auditory cortex, a modality-specific resource. However, in order to analyze one auditory stream in the presence of a simultaneous, competing auditory stream, they suppress the representation of the to-be-ignored stream, which is vying for representation in the same neural area.

### Classifying observer intention from EEG

The attentional modulation of EEG responses was modeled effectively by assuming the ERP response to a mixture of sound streams is a weighted sum of responses to the onsets of the elements in the various streams. Specifically, we modeled the responses to onsets of tones in our sound mixture using ERPs elicited by a single tone presented in quiet, multiplied by a scaling factor that depended on the subject's attention focus. The resulting ERP waveforms accounted for many of the basic features of the total ERP in the selective attention task and much of the variance in the ERP waveform. By comparing the best-fit gains for the left and right streams, we could classify which stream a listener was attending on a single trial basis with an accuracy of 61% (see Figure 8B). Using a more sophisticated cross-correlation template-matching algorithm that weighted the contribution of each electrode based on the variation in ERPs observed during single-note presentations of sound, we were able to achieve even better single-trial classification of attentional focus, near 70%, on average (see Figure 9B).

Our ERP templates were not adapted to individual subject EEG responses; all of our classification results were obtained without taking into account user-specific neural measures (either through cross-subject validation with the measured ERPs, or by assuming that each note in the mixture evoked a stereotypical single-tone ERP when using the 3-component or N1-only component models). Thus, these results demonstrate the feasibility of developing a general-purpose auditory BCI that requires no user-specific training.

### Some caveats and considerations for future work

Although our attention-modulated ERP model fits many aspects of the observed ERPs, we made a number of simplifying assumptions. This simplicity may have allowed us to get robust results, rather than over-fitting our data. Nonetheless, it is important to acknowledge some of the known limitations of our approach.

We did not model the first onset portion of the ERP, since it is a mixture of responses to onsets in all streams, making it impossible to tease apart how each individual stream contributed to the ERP. It is well known that for a single stream in isolation, the first onset is larger than subsequent onsets, something we did not attempt to model here. A more complete model would have to account for stimulus adaptation.

In our analyses, we bandpass filtered the EEG signals and considered signals only in the 2–10 Hz range (delta–theta–alpha band), the frequencies in which the late auditory evoked potential components are strong (P1, N1, and P2). Some past studies suggest that attentional effects are very robust in the theta band (Luo and Poeppel, 2007; Kerlin et al., 2010; Peelle and Davis, 2012). Based on the current results, modulation of onset responses may be a major contributor to these strong attentional effects in the theta frequency region. Indeed, when we modeled the attentional modulation of the N1 response alone, we captured a great deal of the variation in the total ERPs in response to our stimulus mixture. This may be one reason why the N1-only model out-performed the 3-component model. However, there is another alternative explanation; in modeling the total response to our sound stream mixture, we assumed that each note in a given stream (following the initial onset) caused an identical response, of identical magnitude; we then scaled our model single-tone ERP identically for all notes in a given stream. In our 3-component model, this implicitly assumes that attentional modulation scales the P1, N1, and P2 components identically. If the N1 component is modulated more strongly by attention than the other positive components, the N1-only model, which is less able to account for the overall shape of the responses to our 3-s-long stimuli, may nonetheless be better at classifying what stream a listener is attending.

In our modeling, we used the measured ERP to a single tone presented in isolation as the basis for the total ERP in response to every tone onset in every stream. Specifically, we assumed that the onset of every note, from every stream, caused the same stereotypical response, modulo a gain that depended on attention. However, not all stimuli are equally salient, perceptually, something for which the current “onset” model alone cannot account. One might be able to predict differences in stimulus salience by including a more physiologically realistic peripheral processing model (e.g., Rønne et al., 2012) and a computational model of saliency (e.g., Itti and Koch, 2001; Kayser et al., 2005; Kalinli and Narayanan, 2009). Even for stimuli that have similar saliency, different aspects of the ERP (e.g., the latency of the various components) can vary with the stimuli frequency and other aspects of stimulus content (e.g., Scherg et al., 1989). Still, for the limited set of stimuli we used here, our simple model did a good job of capturing key aspects of the EEG response and quantifying how attention modulates this response.

When measuring the single-note ERP, we used a tone complex with 400 Hz fundamental frequency, which was in the middle of the range of fundamental frequencies of the stimuli presented in the main experiment (which had F0s ranging from 240 to 900 Hz). Given that auditory ERPs depend on the spectral content of the sound evoking them (particularly the latency of the N1-component; Roberts et al., 2000; Lütkenhöner et al., 2003), different notes might yield different model parameters when used for the single-note ERP measurement. However, in past reports, latency differences for narrowband notes were on the order of only 10 ms or less, even when the stimulus frequency changed over the range of 200–1000 Hz (Roberts et al., 2000; Lütkenhöner et al., 2003). These small differences are less than one sample for the stimulus sampling rate that we used. In addition, all of our tones were broadband, although they did have different temporal envelopes, as well. While the ERPs might be better fit by accounting for differences in the stimulus notes, we suspect these differences are small.

In the current study, three competing streams were presented, but listeners were only ever asked to attend to the left or right stream. This was done to ensure that the two potential target streams were equally well resolvable in the sensory representation of the stimuli and similar in perceptual saliency. The center stream was included to make the task difficult, thereby making it more likely that listeners would have to sustain attention on the target stream throughout the trial in order to perform the task. We found that the attentional gain to the center stream was uniformly low. Anecdotally, after the end of the experiment, many of our subjects reported that they were completely unaware that there were three streams present; on any given trial, they were conscious of the attended stream, and aware that there was competition from other sounds. While it is not clear from the current study whether listeners could successfully attend to and analyze the center stream in our mixture, pilot experiments show that it is easy to do so. However, further study is needed to explore whether attentional modulation is as strong when listeners are asked to attend to a center stream surrounded by competitors on either side as when they attend streams from the side. Other experiments could be undertaken to map out how many competing simultaneous sources a listener can resolve. It is also not clear how the strength of the attentional modulation that a listener achieves will depend on the spatial position and spectrotemporal content of competing streams (which can affect the ability to segregate sound sources from one another).

Finally, it is worth noting that visual stimuli were only presented during the control (“attend-visual”) condition. It may be that visual-evoked potentials (VEPs) contaminated the observed auditory responses in these trials. Even though the visual arrow onset times were designed to minimize overlap with auditory events (e.g., visual transitions at a given time instant only occurred on some trials, not on every trial, and were themselves temporally isolated from most auditory onsets, etc.), this issue needs to be further clarified by observing VEPs with the visual stimuli presented by themselves.

### Relevance for non-invasive, auditory brain−computer interfaces

In the current study, we purposefully designed the competing streams to have events whose onset responses were temporally resolvable. Moreover, we used the knowledge of the timing of the note onsets in the different streams to fit attentional gains and classify which stream a listener was attending. Having such perfect knowledge about the content and structure of individual streams initially may appear to be a major limitation on how this kind of approach might be implemented in a BCI. However, there are at least a handful of past studies that showed that auditory selective attention can modulate EEG signals enough to allow classification of attentional focus using relatively brief epochs of data (e.g., Kerlin et al., 2010; Hill and Schölkopf, 2012; Lopez-Gordo et al., 2012). These studies typically used competing streams with uncorrelated envelopes (for instance, by using independent streams of ongoing speech; Kerlin et al., 2010; Zion Golumbic et al., 2012). Nearly all BCIs present the human operator with stimuli that are carefully designed to maximize information transfer about the operator's intentions. The current results provide insight into how competing auditory streams might be designed to achieve good single-trial classification, by ensuring that events within each competing stream have onsets that are temporally separated from onsets in the other streams.

Our approach ignored all late components of the ERP, which are associated with higher cognitive processes and decision-making. This may have worked in our study because we designed our behavioral task to force our listeners to wait to make a final judgment about the contour of an attended stream until near the end of the presentation (depending on the stimulus). This likely suppressed later ERP components (like the P3) during the ongoing portion of our stimuli. However, it is worth noting that some recent work using running speech suggests that later components of the EEG response (between 200–220 ms) may be informative about how auditory attention is directed (Power et al., 2012). In addition, many existing EEG BCI systems focus on late ERP components; for instance, there are a number of systems that use the P3 component in response to target and non-target letters to enable users to spell out words (e.g., Farwell and Donchin, 1988; Krusienski et al., 2006; Käthner et al., 2013).

Our approach shows that by carefully designing acoustic stimuli, so that ERPs to events in competing streams can be temporally resolved, attentional modulation of early ERP components is strong and reliable. Given the strength of attentional modulation, our approach should be extendable to track ongoing responses to streams that have temporally uncorrelated structure, rather than requiring events to be completely isolated in time, as long as the competing streams are perceptually segregated (so that the brain can up-regulate the response to the attended stream and suppress the response to ignored streams). Such an approach could lead to a robust, user-friendly auditory-attention driven BCI.

### Conflict of interest statement

The authors declare that the research was conducted in the absence of any commercial or financial relationships that could be construed as a potential conflict of interest.
